# Supplementary material for: Integrative Curriculum Assessment for Inclusion, Representation, and Equity (I-CAIRE)
Source: MedEdPORTAL. 2025 Feb 28;21:11501. doi: 10.15766/mep_2374-8265.11501 (PMC11868286; doi:10.15766/mep_2374-8265.11501)
Supplement: Supplementary file 1 — I-CAIRE Tool.docxScoring Rubric.docx [file mep_2374-8265.11501-s001.zip › B. Scoring Rubric.docx]

**APPENDIX** **B: I-CAIRE Scoring Rubric**

Expert Review Survey for Integrative Curriculum Assessment for Inclusion, Representation, and Equity (I-CAIRE)

Dear Colleague,

We are requesting a few moments of your time to provide an expert review of the I-CAIRE. Because of your expertise in diversity, equity, and inclusion we have identified you as someone whose input would be insightful and valuable to our effort. The purpose of the I-CAIRE is to provide a mechanism for an in-depth review of health professions courses and/or curricula related to health equity, diversity, inclusion, representation, and accessibility. The sections are divided into the following Categories: 1) Health Equity; 2) Curricular Content; 3) Diversity, Inclusion, & Representation; 4) and Accessibility. Each section has a scoring grid to help users understand their relative stage of development in the respective area. The tool includes a glossary to ensure that common language is used to interpret concepts discussed in the tool. The purpose of this study is to establish content validity of the I-CAIRE through expert review.

Q1. Please rate the following Sections 1-4

|  | very high level | high level | fair level | low level | very low level |
| --- | --- | --- | --- | --- | --- |
| Clarity (degree to which section X is clearly defined and understandable) |  |  |  |  |  |
| Comprehensiveness (the degree to which this section includes the primary concepts in this section) |  |  |  |  |  |
| Relevancy (degree to which the survey items in this section are ***appropriate*** and ***connected*** to the concepts in this section) |  |  |  |  |  |
| Validity (the degree to which this section ***captures*** or ***measures*** the concepts as defined at the beginning of this section in the I-CAIRE) |  |  |  |  |  |
| [Free text]  Strengths of section |  | | | | |
| [Free text]  Suggestions for improvement of t section |  | | | | |

Overall, please rate the tool in terms of usability (is the tool user friendly?)

|  | very high level | high level | fair level | low level | very low level |
| --- | --- | --- | --- | --- | --- |
| Overall usability of the tool |  |  |  |  |  |
| Comments: |  |  |  |  |  |
| Would you recommend the I-CAIRE Tool for use at your institution or to another institution? | very likely | likely | maybe | not likely | no way |
|  |  |  |  |  |  |

Please provide any additional comments about the tool in this section.
