# Supplementary material for: Integrative Curriculum Assessment for Inclusion, Representation, and Equity (I-CAIRE)
Source: MedEdPORTAL. 2025 Feb 28;21:11501. doi: 10.15766/mep_2374-8265.11501 (PMC11868286; doi:10.15766/mep_2374-8265.11501)
Supplement: Supplementary file 1 — I-CAIRE Tool.docxScoring Rubric.docx [file mep_2374-8265.11501-s001.zip › A. I-CAIRE Tool.docx]

**APPENDIX A:** Integrative Curriculum Assessment for Inclusion, Representation, and Equity (I-CAIRE) Tool

**Purpose of I-CAIRE**The purpose of the I-CAIRE is to provide a mechanism to conduct an in-depth review of health professions courses and/or curricula related to health equity and representation. This assessment can be helpful in developing new courses or curricula, revising existing courses or curricula, or simply getting some ideas about curricular standards for health equity within health professions education. The I-CAIRE is meant to serve as a catalyst for change and serves an aspirational guide.

**Authors:**

Kupiri Ackerman-Barger, PhD, RN, CNE, ANEF, FAAN, FADLN, Associate Dean for Student Affairs & Health Equity, Diversity and Inclusion, Professor

Elenitsa, M. Sebat, MS, PA-C, Assistant Professor and PhD Student

Jessica, E. Draughon Moret, PhD, RN, Associate Professor

**Beta-testers:**

Amelia Lawless, PhD, LCSW, MPH

Elenitsa, M. Sebat MS, PA-C, PhD Student

Marla Shauer, MSN, RN, CNM, PhD Student

**Expert Reviewers**:

Kenya Beard, EdD, AGACNP-BC, NP-C, CNE, ANEF, FAAN, FADLN

Rosana Gonzalez-Colaso, PharmD, MPH, Assistant Professor

Sheryl Heron, MD, MPH, FACEP, Associate Dean for Community Engagement, Equity and Inclusion and Professor

Nicole Jacobs, PhD, Associate Dean of Diversity, Equity and Inclusion

Gerald Kayingo, PhD, MBA, PA-C, Assistant Dean, Executive Director, and Professor, Author

Robert Lucero, PhD, MPH, RN, FAAN, Associate Dean for Equity, Diversity, and Inclusion and Professor

Lisa Martin, PhD, RN/PHN, AHN-BC, FAAN, Associate Professor, Diversity, Equity and Inclusion Consultant

Rana Halabi Najjar, PhD, RN, CPNP, Associate Professor, Trauma-Informed Educational Practices Consultant

Teresa Thetford, DHSc, MS, PA-C, NP, Physician Assistant Program Director and Associate Professor

Hendry Ton, MD, MS, Associate Vice Chancellor for Health Equity, Diversity, and Inclusion and Professor

**Suggested Citation:** Ackerman-Barger K, Sebat N, Draughon-Moret JE. Integrative Curriculum Assessment for Inclusion, Representation, and Equity Tool (I-CAIRE). *MedEdPORTAL.* 2025;21:11501. <https://doi.org/10.15766/mep_2374-8265.11501>

Table of Contents

[Structure of the I-CAIRE Tool 4](#_Toc177134454)

[Recommendations about How to use the I-CAIRE 4](#_Toc177134455)

[Individual faculty development/Continuous Quality Improvement 5](#_Toc177134456)

[Formal curricular assessment 5](#_Toc177134457)

[Time Commitment 6](#_Toc177134458)

[Integrative Curriculum Assessment for Inclusion, Representation, and Equity (I-CAIRE) 7](#_Toc177134459)

[Section 1: Health Equity 7](#_Toc177134460)

[Section 2: Curriculum Content 8](#_Toc177134461)

[Section 3: Diversity, Inclusion, and Representation 9](#_Toc177134462)

[Section 4: Accessibility 10](#_Toc177134463)

[Glossary 11](#_Toc177134464)

[References: 13](#_Toc177134465)

[Supplement A: Section 1 Health Equity Resources 14](#_Toc177134466)

[Books and Articles 14](#_Toc177134467)

[Indicators for Health, Social Determinants of Health, and Health Equity 15](#_Toc177134468)

[Toolkits and Guides 15](#_Toc177134469)

[Health Equity Training Modules 15](#_Toc177134470)

[Videos and Webinars 16](#_Toc177134471)

[Supplement B: Section 2 Curriculum Content Resources 17](#_Toc177134472)

[Social Justice 17](#_Toc177134473)

[Social Need 17](#_Toc177134474)

[Social Determinants of Health 17](#_Toc177134475)

[Motivational Interviewing 17](#_Toc177134476)

[Trauma-Informed Practices 18](#_Toc177134477)

[Health Equity 18](#_Toc177134478)

[Racism and Anti-racism 18](#_Toc177134479)

[LGBTQ+ care and gender-affirming care 19](#_Toc177134480)

[Implicit Bias and Healthcare Outcomes 20](#_Toc177134481)

[Structural Competencies 21](#_Toc177134482)

[Healthcare provider well-being 21](#_Toc177134483)

[Cultural Humility 22](#_Toc177134484)

[Supplement C: Section 4 Accessibility Resources 22](#_Toc177134485)

# Structure of the I-CAIRE Tool

There are four sections of the I-CAIRE: 1) Social Justice/Health Equity; 2) Curriculum Content; 3) Diversity, Inclusion, & Representation; 4) and Accessibility. None of these domains listed contains an exhaustive list of ways to enhance Inclusion, Representation and Equity in health professions education. We are all continually learning new ways of being. It is our hope that this assessment provides a starting point for changes and discussions within courses and institutions to enhance inclusion excellence and thus health equity.

Each section contains a description followed by a series of statements which are evaluated as to whether there is Integrative, Strong, Minimal, or No Presence. In deciding how to rate specific statements it may be helpful to think about the following definitions:

- Integrative “intending to unify separate concepts or elements”
- Strong “having the power to perform demanding tasks”
- Minimal “of minimum amount, quantity, or degree”^[[1]](#footnote-1)^

There are some items that may be answered objectively – an element is present, or it is absent. The majority of the items rely on subjective understanding, opinions, and situated norms. Therefore, it is crucial to include the rationale for your rating (there is a space provided for this in the survey).

The I-CAIRE is followed by Supplements A-C which include resources specific to each section of the tool.

# Recommendations about How to use the I-CAIRE

Given the importance of inclusion, representation, and equity we highly recommend educators adapt and use the I-CAIRE in any way they feel is helpful to advancing their courses and/or curricula. Below we outline a sample process that we have found helpful to consider while using this tool. Regardless, we encourage educators to view the I-CAIRE as part of an ongoing process of revision and refinement of courses and/or curricula over time.

An assessment without informed action is not enough to create change. Enter the assessment process poised to implement new initiatives and strategies. An assessment alone will not help schools meet their mission and vision. Only thoughtful action can do that. Do not let the I-CAIRE process become a performative effort.

Begin the I-CAIRE process using a growth mindset. Rest assured that all health professions schools are finding their way in diversity, equity, and inclusion. Every school will need to reflect, respond, and grow. There is no pre-designed algorithm or list of steps; each school will be on its unique journey to diversity, equity, and inclusion. But on any journey, we begin with the first steps.

Embrace Humility. As with any assessment, the goal should be to get the process right, not to be right. Be willing to take an honest look at how your school operates. This means you note both deficiencies and achievements. When you find a deficiency, be courageous in responding to it in a way that aligns with your mission and vision. When you find areas of success, consider sharing those efforts with others so that we can learn and grow together.

Include Multiple Perspectives

Remember that multiple perspectives will provide a more comprehensive view of your curriculum. Diverse viewpoints are associated with better problem-solving and innovation. Ideally the I-CAIRE process would be a collaborative process conducted by a diverse and representative group of school members committed to upholding the school’s mission and vision for diversity, equity, and inclusion. When forming the group(s) of people to conduct the I-CAIRE ask if any groups are missing from the discussion. Is there a place for students in the conversation? Community members? Educational staff?

## Individual faculty development/Continuous Quality Improvement

The instructor of record may conduct a self-assessment of any of their courses using the I-CAIRE at any time. We believe that any starting point is a great one. Educators may choose to review their course in light of all four section of the I-CAIRE. Alternatively, they may select one section and start their self-assessment and continuous course improvement focusing only on the statements and resources for that section.

## Formal curricular assessment

Step 1: Select your team. An ideal team would include a minimum of 2-3 people with diverse identities (e.g., race, gender, age, sexuality, class, national origin) and roles (program leaders, faculty, and/or staff) who work together to evaluate the course or curriculum.  As part of a formal curricular assessment, we encourage the instructor of record for a particular course be a part of the evaluation team. Self-assessment from the instructor of record would facilitate timely evaluation. Similarly, when opportunities for growth are identified, the instructor of record would best determine which resources to use, and optimal implementation. Evaluators would ideally have received training on diversity, equity, and inclusion with a special emphasis on concepts of health equity, diversity, and representation.  Please see glossary for terms.

Step 2: Select the courses/components and/or lessons to analyze. If your institution plans to create an ongoing assessment process, we recommend rotating the courses each year so that the curriculum is constantly undergoing evaluation and to keep the workload at a manageable level.

Step 3: Review the I-CAIRE. Ensure each member of the curriculum assessment team understands the terminology (see glossary) and the key concepts of measure in each section.

Step 4: Conduct the curriculum assessment. Document rationales for ratings and initial ideas for incorporating Inclusion, Representation and Equity.

Step 5: Review and collate ratings and rationales. Collaboratively identify key areas for growth. Use the included resources (Supplements A-C) as a starting point for further integrating Inclusion, Representation and Equity into your course and/or curriculum.

Step 6: Report back on recommendations. We would like to reiterate that this assessment is meant to be transformational and aspirational, not punitive.

# Time Commitment

The amount of time spent on this activity will depend on the goal of the user. A self-assessment of a course by the instructor could be performed in approximately one hour. A course assessment by non-instructor evaluator with full access to the course would likely take about two hours per evaluator depending on course content organization. The time investment to complete assessments for an entire program would be significantly increased. We recommend that program assessments occur over a period time rather than all at once.

To approximate the time commitment for evaluating an entire program multiple the total number of courses to be evaluated by the number of instructor self-assessment hours (1 hour per course) and then add the number of courses to be evaluated multiplied by non- instructor assessments (2 hours per course). For example, if school A has 10 courses and will conduct self-assessments and two non-course evaluator assessment per course: 10 courses x 1 hour = 10 hours and 10 courses x 4 hours= 40 hours. The total number of hours in this case would be 50 collective hours.

#

# Integrative Curriculum Assessment for Inclusion, Representation, and Equity (I-CAIRE)

## Section 1: Health Equity

This section addresses the degree to which courses or curricula teach health equity. Health equity refers to a health outcome that is achieved with the elimination of health disparities. Health disparities within the U.S. health system are historic, pernicious and can be considered complex problems in which many perspectives, insights and innovations will be needed for change. This section addresses how well we poise students graduating from our program to address health disparities and promote health equity. See SUPPLEMENT A for Resources.

- Integrative “intending to unify separate concepts or elements”
- Strong “having the power to perform demanding tasks”
- Minimal “of minimum amount, quantity, or degree”

*Is there evidence that course materials and instructional activities align with the statements below?*

| Statements | Integrative  Presence | Strong  Presence | Minimal  Presence | No  Presence |
| --- | --- | --- | --- | --- |
| Course materials include student learning objectives related to health equity. |  |  |  |  |
| The course materials and instructional activities promote or provoke critical questions about social determinants of health, health inequities and health disparities. |  |  |  |  |
| The course materials and instructional activities allow students to explore the relationship between socio/political, economic and/or environmental factors related to health and well-being. |  |  |  |  |
| The course materials provide avenues for students to see how healthcare professionals can take actions that combat inequity and promote health equity. |  |  |  |  |
| The course materials reflect that health outcomes are the result of health inequities embedded within societal and/or systemic context rather than any assumption of biological inferiority or simply the result of choices of individuals. |  |  |  |  |
| The course materials encourage students to critically examine power structures and ethical practices in healthcare. |  |  |  |  |
| The course learning assessment and evaluation addresses student learning related to health equity. |  |  |  |  |

## Section 2: Curriculum Content

There is course/curricular core content that students need to develop the knowledge and skills to become equity-minded and to promote health equity. There are several domains in this section that have been identified by the National Academies of Sciences, Engineering, and Medicine (2021) as essential educational content in health professions to meet the challenge of addressing the social determinants of health, advancing health equity, and improving population health. See SUPPLEMENT B for Resources

- Integrative “intending to unify separate concepts or elements”
- Strong “having the power to perform demanding tasks”
- Minimal “of minimum amount, quantity, or degree”

*There is evidence of the following in the course content:*

| Topics* | Integrative  Presence | Strong  Presence | Minimal  Presence | No Presence |
| --- | --- | --- | --- | --- |
| Social Justice |  |  |  |  |
| Social Need |  |  |  |  |
| Social Determinants of Health |  |  |  |  |
| Population Health |  |  |  |  |
| Environmental Health |  |  |  |  |
| Motivational Interviewing |  |  |  |  |
| Trauma-informed care |  |  |  |  |
| Health Equity |  |  |  |  |
| Racism and Anti-racism |  |  |  |  |
| LGBTQ+ care and gender-affirming care |  |  |  |  |
| How implicit or unconscious bias can impact care (areas should include, but are not limited to race, gender, sexual orientation, age, ability, religion, language, immigration status, weight, mental health, homeless, and substance use disorder) |  |  |  |  |
| Structural competencies such as structural racism, structural oppression, structural violence. |  |  |  |  |
| LGBTQ+ care and gender-affirming care |  |  |  |  |
| Healthcare provider well-being |  |  |  |  |
| Cultural Humility |  |  |  |  |
| Culturally and Linguistically Appropriate Services Standards |  |  |  |  |

******Please see the glossary for definitions of these terms.*

## Section 3: Diversity, Inclusion, and Representation

Diversity represents a driver of academic and institutional excellence. Diverse health professions teams may be better equipped to solve complex health problems and address health disparities because they tend to be interested in solving health inequities and bring a wealth of knowledge and perspectives to the problem-solving process (Swartz, Palermo, Masur & Aberg, 2019). However, to leverage the power of diversity we must also be inclusive. In this section we evaluate course materials and resources (PowerPoints, cases, textbooks, exams, learning management sites, etc.) to ensure they reflect diversity, are inclusive, and portray positive and accurate representations of communities of underrepresented people without stereotyping. Consider representation in terms of national, state, and a local demographics and communities. See SUPPLEMENT A-C for Resources.

- Integrative “intending to unify separate concepts or elements”
- Strong “having the power to perform demanding tasks”
- Minimal “of minimum amount, quantity, or degree”

*There is evidence of the following in the course content:*

| Statements | Integrative  Presence | Strong  Presence | Minimal Presence | No  Presence |
| --- | --- | --- | --- | --- |
| The course materials reflect diverse, inclusive, and representative groups of people. |  |  |  |  |
| The course materials reflect both the strengths of communities represented as well as the challenges they experience. |  |  |  |  |
| The course materials acknowledge and/or incorporate the expertise and wisdom of underrepresented communities |  |  |  |  |
| The course materials have images, names, scenarios and text that holistically reflect the experiences, interests, strengths and challenges of underrepresented groups. These materials are presented without stereotyping. |  |  |  |  |
| Issues of social justice, equity, anti-racism, experiences, and contributions of people from historically marginalized communities are reflected in homework/classroom assignments and assessments. |  |  |  |  |
| The resources, materials, and technology referenced in lessons include resources from historically marginalized communities. |  |  |  |  |

## Section 4: Accessibility

In alignment with a commitment to excellence in teaching and learning as well as our commitment to diversity, equity and inclusion schools must ensure that their teaching and course materials (PowerPoints, cases, textbooks, exams, learning management systems, etc.) are accessible to a broad range of learners. See Supplement C for Resources.

- Integrative “intending to unify separate concepts or elements”
- Strong “having the power to perform demanding tasks”
- Minimal “of minimum amount, quantity, or degree”

*There is evidence that the course materials are designed to appeal to a broad array of learners.*

| Statements | Integrative  Presence | Strong  Presence | Minimal Presence | No Presence |
| --- | --- | --- | --- | --- |
| This course incorporates inclusive teaching and learning approaches that are designed to include and respond to the diverse experiences of students in the course. |  |  |  |  |
| There are a mix of educational approaches applied in this course. |  |  |  |  |
| Tests and other learning assessments use multiple measurements (multiple-choice, essay, short answer, performance, presentations, etc.) |  |  |  |  |

*There is evidence that course materials were designed to be accessible for students with DiversABILITIES.*

| Statements | Integrative Presence | Strong Presence | Minimal Presence | No Presence |
| --- | --- | --- | --- | --- |
| The syllabus and/or learning management gives clear direction to students about how to seek accommodations in the classroom. |  |  |  |  |
| Videos are properly captioned |  |  |  |  |
| Auto-captioning is used in Zoom (or virtual platforms) |  |  |  |  |
| Microphones are used in large classrooms and/or rooms with poor acoustics |  |  |  |  |
| PowerPoints and class materials are available to students to review prior to class. |  |  |  |  |
| Lecture materials are recorded for students to review. |  |  |  |  |
| Coloring of text accommodates students who may be colorblind. For example, if using colors use bold contrasting colors. Avoid us of green or red font. |  |  |  |  |
| There is a mechanism in the course for students to provide feedback on accessibility. |  |  |  |  |

# Glossary

**Anti-racism** refers to taking a committed stand against racism, a stand that translates into ACTION that interrupts racism in all of its forms whether personal or institutional, blatant or routine, intended or unintended (Trepganier, 2007).

**Cultural Humility** “Cultural humility in clinical practice is best defined not by a discrete endpoint [e.g. ‘competence’] but as a commitment and active engagement in a lifelong process that individuals enter into on an ongoing basis with patients, communities, colleagues, and with themselves.” ([Tervalon & Murray-Garcia, 1998, p.118](https://melanietervalon.com/wp-content/uploads/2013/08/CulturalHumility_Tervalon-and-Murray-Garcia-Article.pdf)).

**DiversABILITY** There are many differing abilities that have often been called “disabilities”. The term diversABILITY reframes how we think about abilities and values the skills and perspectives that people with diverse abilities bring to the learning environment and workplace.

**Environmental Health** Focuses on the relationship between people and their environment. Please see: https://www.neha.org/about-neha/definitions-environmental-health for more information.

**Equity-minded** refers to a mode of thinking exhibited by practitioners who are willing to assess their own racialized assumptions, to acknowledge their lack of knowledge in the history of race and racism, to take responsibility for the success of historically underserved and minoritized groups, and to critically assess racialization in their own practices (McNair, Bensimon, Malcom-Piqueux, 2020, p. 20).

**Health disparities** exist when a health outcome “is seen to a greater or lesser degree between populations.” They are “particular types of health differences that are closely linked with social, economic, and/or environmental disadvantage. Health disparities adversely affect groups of people who have systematically experienced greater obstacles to health based on their racial or ethnic group; religion; socioeconomic status; gender; age; mental health; cognitive, sensory, or physical disability; sexual orientation or gender identity; geographic location; or other characteristics historically linked to discrimination or exclusion ([Healthy People 2020](https://www.healthypeople.gov/2020/about/foundation-health-measures/Disparities)).”

**Health equity** “Achieving heath equity requires valuing all individuals and populations equally, recognizing and rectifying historical injustices, and providing resources according to need. Health disparities will be eliminated when health equity is achieved” (Jones, 2014).

**Health inequities** are systemic, avoidable, and unjust social and economic policies and practices that create barriers to opportunities and resources which can, ultimately, lead to health disparities.

**Historically marginalized groups** refers to groups who have been historically and continue to be underrepresented, underserved and who have been deemed not as important or valued within a society.

**Population Health** At its core, population health views the “population” as the unit of care.

**Social Justice** The Latin root of the word social means friend, ally and represents people or social units. The word justice is derived from the Old French and has to do with rights or allocation of resources. Social Justice then addresses fair and just allocation of resources among groups or people.

**Social Determinants of Health** can be defined as factors that contribute to health or illness. These can include the environments in which people are born, grow, live, learn, work, play, worship, and age that affect a wide range of health, functioning, and quality of life outcomes and risks [(Healthy People 2020)](https://www.healthypeople.gov/2020/topics-objectives/topic/social-determinants-of-health). Understanding health disparities can provide vital contextual information about health disparities

**Social Need** Unmet social needs contribute to disparate health outcomes. If the health outcome is what we see during a patient visit, the social need can become more apparent through social needs screening where it can become apparent that an individual, family or community lacks access to a resource (food, transportation, ability to pay for medication, etc.) that would otherwise contribute to a better health outcome. Understanding social need can help us understand what a patient, family, or community needs, but we also need to understand how these needs manifest and how they determine health.

**Trauma-Informed Care** In plain language, providers cannot know whether an individual patient has experienced a traumatic event or series of events just by looking at them. Trauma is pervasive, and therefore treating every patient as if they have experienced trauma at some point allows for non-harmful patient-centered care. The Substance Abuse Mental Health Services Administration defines Trauma Informed Care as: “A program, organization, or system that is trauma-informed realizes the widespread impact of trauma and understands potential paths for recovery; recognizes the signs and symptoms of trauma in clients, families, staff, and others involved with the system; and responds by fully integrating knowledge about trauma into policies, procedures, and practices, and seeks to actively resist re-traumatization.”

Please see: https://ncsacw.samhsa.gov/userfiles/files/SAMHSA_Trauma.pdf for additional information.

# References:

Jones, C. (2014). *Systems of power, axes of inequity: parallels, intersections, braiding the strands.* Medical Care, 52(12). S71-S75.

Healthy People 2020. Retrieved from <https://www.healthypeople.gov/2020/>

McNair, T., Bensimon, E.M., Malcom-Piqueux, L. (2020). *From equity talk to equity walk.* Hoboken, NJ: Jossey-Bass.

National Academies of Sciences, Engineering, and Medicine 2021. *The Future ofNursing 2020-2030: Charting a Path to Achieve Health Equity*. Washington, DC:The National Academies Press. <https://doi.org/10.17226/25982>.

New England Resource Center for Higher Education (2016). *NERCHE Self-Assessment Rubric for the Institutionalization of Diversity, Equity, and Inclusion in Higher Education.*<https://www.wpi.edu/sites/default/files/Project_Inclusion_NERCHE_Rubric-Self-Assessment-2016.pdf>

Peoples, L.Q., Islam, T., & Davis, T. (2021). *The culturally responsive-sustaining STEAM curriculum scorecard.* New York: Metropolitan Center for Research on Equity and the Transformation of Schools, New York University

Substance Abuse Mental Health Services Administration. (2014). *SAMHSA’s concept of trauma and guidance for a trauma-informed approach.* U.S. Department of Health and Human Services. Retrieved from <https://ncsacw.samhsa.gov/userfiles/files/SAMHSA_Trauma.pdf>.

Swartz, T. H., Palermo, A. S., Masur, S. K., & Aberg, J. A. (2019). The Science and Value of Diversity: Closing the Gaps in Our Understanding of Inclusion and Diversity. The Journal of infectious diseases, 220(220 Suppl 2), S33–S41. <https://doi.org/10.1093/infdis/jiz174>

Trepganier, B. (2007). *Silent Racism.* New York, NY: Routledge.

University of California Davis (n.d.) The principles of community. Retrieved on Mar. 4, 2022 from <https://diversity.ucdavis.edu/principles-community>.

# Supplement A: Section 1 Health Equity Resources

## Books and Articles

Ackerman-Barger, K. (2022). Advancing health equity: The rise of equity-minded nurses. Campaign for Action. <https://campaignforaction.org/the-rise-of-equity-minded-nurses/>

Braveman, P., Arkin, E., Orleans, T., Proctor, D., Plough, A. (2017). [What is health equity? Robert Wood Johnson](https://www.rwjf.org/en/library/research/2017/05/what-is-health-equity-.html) [Foundation](https://www.rwjf.org/en/library/research/2017/05/what-is-health-equity-.html). <https://www.rwjf.org/en/library/research/2017/05/what-is-health-equity-.html>

Brockie, T.N., Heinzelmann, M., & Gill, J. (2010). A framework to examine the role of epigenetics in health disparities among Native Americans. *Nursing Research and Practice* (2013), 1-9.

Castrucci, B.C. & Auerbach, J. (2019). Meeting individual social needs falls short of addressing social determinants of health. Health Affairs. DOI: 10.1377/hblog20190115.234942. Retrieved from https://[www.healthaffairs.org/](http://www.healthaffairs.org/) do/10.1377/hblog20190115.234942/full/

Compton, M. & Shim, R. (2015). The Social Determinants of Mental Health. Arlington, VA: American Psychiatric Publishing.

Crear-Perry, J., Correa-de-Araujo, R., Lewis Johnson, T., McLemore, M.R., Neilson, E., & Wallace, M. (2021). Social and structural determinants of health inequities in maternal health. *Journal of Women’s Health*. 230-235

.

Dawes, D. E. (2020). The Political Determinants of Health. Baltimore, MD: Johns Hopkins University.

Geronimus, A. (2023). *Weathering: The Extraordinary Stress of Ordinary Life in an Unjust Society*. New York, NY: Little, Brown and Company.

Hassmiller, S. (2019). [Perfectly positioned: galvanizing nurses to address the social determinants of health.](https://www.healthaffairs.org/do/10.1377/forefront.20190429.781982/full/) *Health Affairs.*

Hassmiller, S. & Daniel, G.A. (2023). Taking action: Top 10 priorities to promote health equity and well-being in nursing. Indianapolis, IN: Sigma Theta Tau International.

Jones, C. (2014). Systems of power, axes of inequity: parallels, intersections, braiding the strands. Medical Care, 52(12). S71-S75.

National Academies of Sciences, Engineering, and Medicine (2021). The Future of Nursing 2020-2030: Charting a Path to Achieve Health Equity. Washington, DC: The National Academies Press

Olson, D. P., Oldfield, B. J., & Morales Navarro, S. (2019, March 18). [Standardizing social determinants of health](https://www.healthaffairs.org/do/10.1377/forefront.20190311.823116/full/) [assessments](https://www.healthaffairs.org/do/10.1377/forefront.20190311.823116/full/). *Health Affairs Blog.*

Thornton, R. L., Glover, C. M., Cené, C. W., Glik, D. C., Henderson, J. A., & Williams, D. R. (2016). [Evaluating strategies](https://www.ncbi.nlm.nih.gov/pmc/articles/PMC5524193/) [for reducing health disparities by addressing the social determinants of health. *Health Affairs,*](https://www.ncbi.nlm.nih.gov/pmc/articles/PMC5524193/) 35(8), 1416-1423.

Williams, D. R. (2020). [Why Discrimination Is a Health Issue](https://www.rwjf.org/en/blog/2017/10/discrimination-is-a-health-issue.html). RWJF.

Yudell, M., Roberts, D., DeSalle, R., Tishkoff, S. (2016) Taking race out of human genetics: Engaging a century-long debate about the role of race in science. *Science,* 6273, 564-565.

## Indicators for Health, Social Determinants of Health, and Health Equity

- [RWJF County Health Rankings](http://www.countyhealthrankings.org/)
- [Healthy People 2030-Social Determinants of Health](https://health.gov/healthypeople/priority-areas/social-determinants-health)
- [CDC’s Social Vulnerability Index](https://svi.cdc.gov/Documents/FactSheet/SVIFactSheet.pdf) (SVI)
- [Introduction to CDC's SVI](https://www.youtube.com/watch?reload=9&v=u5m0Lb3B4UY&feature=youtu.be) (Video)
- [Methods for CDC's SVI](https://www.youtube.com/watch?v=REKFHOryflA&feature=youtu.be) (Video)
- [City Health Dashboard](https://www.cityhealthdashboard.com/)
- [AARP Livability Index](https://livabilityindex.aarp.org/faqs#faq-1)
- [Feeding America: Working together to end hunger](https://www.feedingamerica.org/our-work/fighting-to-end-hunger)
- [Feeding America: Taking Action to help hardworking families](https://www.feedingamerica.org/take-action)
- [Food Research & Action Center—a national nonprofit organization working to eliminate poverty-related hunger and undernutrition in the U.S.](http://frac.org/)
- [Social Missions Metrics Initiative-Mullan Institute](https://socialmissionmetrics.gwhwi.org/#:~:text=The%20Social%20Mission%20Metrics%20Initiative,mission%20in%20health%20professions%20education.)
- [Association of American Medical Colleges Center for Health Justice](https://www.aamchealthjustice.org/)

## Toolkits and Guides

- [Building Coalitions to Promote Health Equity: A Toolkit for Action](https://campaignforaction.org/wp-content/uploads/2021/05/AARP_CCNA_HealthEquityToolkit_041522_111.pdf)
- [Tribal Equity Toolkit](https://www.thetaskforce.org/wp-content/uploads/2014/09/TET3.0.pdf)
- [The CDC Community Health Assessment and Group Evaluation (CHANGE) Data-collection Tool and Planning Resource](https://www.cdc.gov/nccdphp/dnpao/state-local-programs/change-tool/index.html)
- [CDC Health Equity Resource Toolkit for State Practitioners Addressing Obesity Disparities](https://www.cdc.gov/obesity/downloads/CDCHealthEquityObesityToolkit508.pdf)
- [Policy resources to support SDOH](https://www.cdc.gov/socialdeterminants/policy/index.htm)
- [The Community Guide: Develop Evidence-Based Policies](https://www.thecommunityguide.org/about/policy-development)

## Health Equity Training Modules

- [Prevention Institute: Health Equity Training Series](http://www.preventioninstitute.org/sites/default/files/publications/HD_training_series_1pgr_FINAL.pdf)
- [Addressing Health Equity: A Public Health Essential](https://phtc-online.org/learning/?courseId=41) (course with continuing education credits)
- [Health Equity in Public Policy: Messaging Guide for Policy Advocates](https://voicesforhealthykids.org/assets/resources/health-equity-messaging-guide-1600967742.pdf)

## Videos and Webinars

- [How Unequal Opportunity Created Unequal Health: Interview of David Williams by Michelle Goodwin.](https://www.youtube.com/watch?v=sJ6PgT3qrJ8) (Jan. 2022). The Hastings Center Health Equity Summit “Righting the Wrongs, Tackling Health Inequities”. (58:53 minutes)
- [To Improve Health Equity, Look at Politics: Interview of Daniel Dawes by Phillip Alberti.](https://www.thehastingscenter.org/news/to-improve-health-equity-look-at-politics/) (Jan. 2022). The Hastings Center Health Equity Summit “Righting the Wrongs, Tackling Health Inequities”. (53:55 minutes)
- (Jan. 2022). The Hastings Center Health Equity Summit “Righting the Wrongs, Tackling Health Inequities”.
- (Jan. 2022). The Hastings Center Health Equity Summit “Righting the Wrongs, Tackling Health Inequities”.
- Hastings Center Webinars on Health Justice List <https://www.thehastingscenter.org/webinars/>

# Supplement B: Section 2 Curriculum Content Resources

## Social Justice

Valderama-Wallace, C. P., & Apesoa-Varano, E. C. (2019). "Spinning Their Wheels … "-Influences That Shape How Nurse Educators Teach Social Justice. *Policy, politics & nursing practice*, *20*(4), 239–251. https://doi.org/10.1177/1527154419881726

## Social Need

Byhoff, E., & Gottlieb, L. M. (2022). When there is value in asking: an argument for social risk screening in clinical practice. *Annals of Internal Medicine*, *175*(8), 1181–1182.

Castrucci, B.C. & Auerbach, J. (2019). Meeting individual social needs falls short of addressing social determinants of health. Health Affairs. DOI: 10.1377/hblog20190115.234942. Retrieved from <https://www.healthaffairs.org/>

do/10.1377/hblog20190115.234942/full/

Billioux, A., Verlander, K., Anthony, S., Alley, D., et al. (2017). Standardized Screening for Health-Related Social Needs in Clinical Settings: The Accountable Health Communities Screening Tool. *NAM Perspectives*, *7*(5).

Garg, A., Boynton-Jarrett, R., & Dworkin, P. H. (2016). Avoiding the unintended consequences of screening for social determinants of health. *The Journal of the American Medical Association*, *316*(8), 813–814.

O’Gurek, D. T., & Henke, C. (2018). A practical approach to screening for social determinants of health. *Family practice management*, *25*(3), 7–12.

Olson, D. P., Oldfield, B. J., & Morales Navarro, S. (2019, March 18). [Standardizing social determinants of health](https://www.healthaffairs.org/do/10.1377/forefront.20190311.823116/full/) [assessments](https://www.healthaffairs.org/do/10.1377/forefront.20190311.823116/full/). *Health Affairs Blog.*

The Health Leads Screening Toolkit <https://healthleadsusa.org/resources/the-health-leads-screening-toolkit/>

## Social Determinants of Health

See Supplement A: Section 1 resources.

## Motivational Interviewing

Spirit of Motivation Interviewing (video) (6:12 minutes) <https://www.youtube.com/watch?v=APPoKvTPhog>

Bennet, M. (2017). *Connecting paradigms: A trauma-informed & neurological framework for motivational interviewing implementation.* Denver: CO: Bennet Innovation Group.

Hettema, J., Steele, J., & Miller, W. R. (2005). Motivational interviewing. *Annual Review of Clinical Psychology*, *1*, 91–111.

Noonan, W. C., & Moyers, T. B. (1997). Motivational interviewing. *Journal of Substance Misuse*, *2*(1), 8–16.

## Trauma-Informed Practices

Felitti, V.J., Anda, R.F., Nordenberg, D., Williamson, D.F., Spitz, A.M., Edwards, V... Marks, J. (1998). Relationship of childhood abuse and household dysfunction to many of the leading causes of death in adults: The adverse childhood experiences (ACE) study. *American Journal of Preventive Medicine,* 14, 245-248.

Goldstein, E., Murray-García, J., Sciolla, A. F., & Topitzes, J. (2018). Medical Students' Perspectives on Trauma-Informed Care Training. The Permanente journal, 22, 17–126. https://doi.org/10.7812/TPP/17-126

Harris, N.B. (2018). The deepest well: Healing the long-term effects of childhood adversity. New York, NY: Houghton Mifflin Harcourt

Mate’,G. (2022). The myth of normal: trauma, illness & healing in a toxic culture. New York, NY: Penguin Random House.

Van Der Kolk, B. (2014). The body keeps the score” Brain, mind, and body in the healing of trauma. New York, NY: Penguin Books.

Najjar, R.H. (2023) A trauma-informed approach provides a framework for achieving health equity. <https://campaignforaction.org/trauma-informed-approach-provides-framework/>

Najjar, R. & Ackerman-Barger, K. (2024, August). Advancing equity and justice in academic nursing through trauma-informed educational practices. Journal of Nursing Education. 63 (8) 507-514

Sciolla, A.F. (2017). An Overview of Trauma-Informed Care. In: Eckstrand, K., Potter, J. (eds) Trauma, Resilience, and Health Promotion in LGBT Patients. Springer, Cham. <https://doi.org/10.1007/978-3-319-54509-7_14>

Venet, A.S. (2021). Equity-Centered Trauma-Informed Educational Practices. New York, NY: W.W. Norton & Company

## Health Equity

See Supplement A: Section 1 resources.

## Racism and Anti-racism

Balch, B. (2020). [**Curing health care of racism: Nikole Hannah-Jones and Ibram X. Kendi, PhD, call**](https://www.aamc.org/news-insights/curing-health-care-racism-nikole-hannah-jones-and-ibram-x-kendi-phd-call-institutions-foster-change) [**on institutions to foster change**](https://www.aamc.org/news-insights/curing-health-care-racism-nikole-hannah-jones-and-ibram-x-kendi-phd-call-institutions-foster-change). Association of American Medical Colleges.

Brockie, T.N., Heinzelmann, M., & Gill, J. (2010). A framework to examine the role of epigenetics in health disparities among Native Americans. *Nursing Research and Practice* (2013), 1-9.

Compton, M.T., and Shim, R.S. (2015). *The social determinants of mental health*. Arlington, VA: American Psychiatric Publishing.

Ford, C.L., Griffith, D.M., Bruce, M.A., Gilbert, K.L. (2019). Racism: Science & tools for the public health professional. Washington, DC: APHA Press.

Fletcher, F. E., Ray, K.S., Brown, V.A., Smith, P.T. (2022). Addressing Anti-Black Racism in Bioethics: Responding to the Call. Hastings Center Report.

Hogarth, R.A. (2017). Medicalizing blackness: Making racial difference in the Atlantic world 1780-1840. Chapel Hill, NC: University of North Carolina.

[**Human Genome Project**](https://www.genome.gov/human-genome-project).

Jones, C. P. (2000). Levels of racism: A theoretic framework and a gardener’s tale. American Journal of Public Health, 90, 1212-1215.

Jones, C. (2014). Systems of power, axes of inequity: parallels, intersections, braiding the strands. Medical Care, 52(12). S71-S75.

Kendi, I. X. (2019). How to be an anti-racist. New York, NY: Penguin Random House.

McGhee, H. (2021). The sum of us: What racism costs everyone and how we can prosper together. New York, NY: Penguin Random House.

MedEdPortal Antiracism: <https://www.mededportal.org/anti-racism>

Moscou, S., Baker, S.(2019) The role of race in clinical decision making. *Nurse Practitioner,* 43(3), 41-46.

Roberts, D. (2008). Is Race-Based Medicine Good for Us? African American Approaches to Race, Biomedicine, and Equality. *The Journal of Law, Medicine & Ethics,* 36(3), 537-545.

[**University of California San Francisco. Anti-Racism Resources**](https://diversity.ucsf.edu/programs-resources/training/antiracism-training).

Washington H. (2006). Medical apartheid: The dark history of medical experimentation n Black Americans from colonial times to present. New York, NY: Random House.

Yudell, M., Roberts, D., DeSalle, R., Tishkoff, S. (2016) Taking race out of human genetics: Engaging a century-long debate about the role of race in science. *Science,* 6273, 564-565.

## LGBTQ+ care and gender-affirming care

Coleman, E., Radix, A. E., Bouman, W.P., Brown, G.R., de Vries, A. L. C., Deutsch, M. B., Ettner, R., Fraser, L., Goodman, M., Green, J., Hancock, A. B., Johnson, T. W., Karasic, D. H., Knudson, G. A., Leibowitz, S. F., Meyer-Bahlburg, H. F.L., Monstrey, S. J., Motmans, J., Nahata, L., ... Arcelus, J. (2022). Standards of Care for the Health of Transgender and Gender Diverse People, Version 8. International Journal of Transgender Health, 23(S1), S1-S260.

<https://doi.org/10.1080/26895269.2022.2100644>

Deutsch, M. B., Bowers, M. L., & Radix, A. (2019). Transgender medical care in the United States: A historical perspective. *The GLMA handbook …*.

Frost, D. M., & Meyer, I. H. (2023). Minority stress theory: application, critique, and continued relevance. *Current opinion in psychology*, 101579.

GLMA: Health Professionals Advancing LGBTQ+ Equality (previously known as the Gay & Lesbian Medical Association) <https://www.glma.org/>

Hafeez, H., Zeshan, M., Tahir, M. A., Jahan, N., & Naveed, S. (2017). Health care disparities among lesbian, gay, bisexual, and transgender youth: A literature review. *Cureus*, *9*(4), e1184.

Harris, M. (2023) Gender-Affirming Nursing is Equity-Minded Nursing A trauma-informed approach provides a framework for achieving health <https://campaignforaction.org/gender-affirming-nursing-is-equity-minded-nursing/>

Medina-Martínez, J., Saus-Ortega, C., Sánchez-Lorente, M. M., Sosa-Palanca, E. M., García-Martínez, P., & Mármol-López, M. I. (2021). Health inequities in LGBT people and nursing interventions to reduce them: A systematic review. *International Journal of Environmental Research and Public Health*, *18*(22).

Meyer, I. H., & Frost, D. M. (2012). Minority stress and the health of sexual minorities. In C. J. Patterson & A. R. D’Augelli (Eds.), *Handbook of psychology and sexual orientation* (pp. 252–266). Oxford University Press.

Mitchell, A. & Somers, M. (2018). Healthcare professionals working with LGBTQ Patients. *Journal of Advances in Medicine and Medical Education. 25* (12), 1-5.

## Implicit Bias and Healthcare Outcomes

Chapman, E. N., Kaatz, A., & Carnes, M. (2013). Physicians and implicit bias: how doctors may unwittingly perpetuate health care disparities. *Journal of General Internal Medicine*, *28*(11), 1504–1510.

Dehon, E., Weiss, N., Jones, J., Faulconer, W., Hinton, E., & Sterling, S. (2017). A systematic review of the impact of physician implicit racial bias on clinical decision making. *Academic Emergency Medicine*, *24*(8), 895–904.

Fanta, M., Ladzekpo, D., & Unaka, N. (2021). Racism and pediatric health outcomes. *Current problems in pediatric and adolescent health care*, *51*(10), 101087.

Greenwood, B. N., Hardeman, R. R., Huang, L., & Sojourner, A. (2020). Physician-patient racial concordance and disparities in birthing mortality for newborns. *Proceedings of the National Academy of Sciences of the United States of America*, *117*(35), 21194–21200.

Guglielminotti, J., Samari, G., Friedman, A. M., Lee, A., Landau, R., & Li, G. (2022). Nurse workforce diversity and reduced risk of severe adverse maternal outcomes. *American Journal of Obstetrics & Gynecology MFM*, *4*(5), 100689.

Hall, W. J., Chapman, M. V., Lee, K. M., Merino, Y. M., Thomas, T. W., Payne, B. K., Eng, E., et al. (2015). Implicit racial/ethnic bias among health care professionals and its influence on health care outcomes: A systematic review. *American Journal of Public Health*, *105*(12), e60-76.

Noone, J., & Najjar, R. H. (2021). Minimizing unconscious bias in nursing school admission. *The Journal of nursing education*, *60*(6), 317–323.

Persaud, S. (2019). Addressing unconscious bias: A nurse leader’s role. *Nursing administration quarterly*, *43*(2), 130–137.

Ross, H. (2014). *Everyday bias: Identifying and navigating unconscious judgements in our daily lives.* London, England: Rowman & Littlefield.

Thirsk, L. M., Panchuk, J. T., Stahlke, S., & Hagtvedt, R. (2022). Cognitive and implicit biases in nurses’ judgment and decision-making: A scoping review. *International Journal of Nursing Studies*, *133*, 104284.

## Structural Competencies

Hansen, H., & Metzl, J. (2016). Structural competency in the U.S. healthcare crisis: putting social and policy interventions into clinical practice. *Journal of bioethical inquiry*, *13*(2), 179–183.

Metzl, J.M. & Hansen, H. (2014). Structural competence: Theorizing a new medical engagement with stigma and inequality. *Social Science & Medicine,* 103, 126-133.

Metzl, J. M., & Petty, J. (2017). Integrating and assessing structural competency in an innovative prehealth curriculum at vanderbilt university. *Academic Medicine*, *92*(3), 354–359.

Neff, J., Knight, K. R., Satterwhite, S., Nelson, N., Matthews, J., & Holmes, S. M. (2017). Teaching structure: A qualitative evaluation of a structural competency training for resident physicians. *Journal of General Internal Medicine*, *32*(4), 430–433.

## Healthcare provider well-being

Hammond, C. (2019). The art of rest: How to find respite in the modern age. Edinburgh, United Kingdom

Lipsky, L. (2009). Trauma stewardship: An everyday guide to caring for self while caring for others. Oakland, CA: Berrett-Koehler

Nagoski, E. & Nagoski A. (2020). Burnout: The secret to unlocking the stress cycle. New York, NY: Penguin Random House.

Parker, G. (2020). Restorative yoga for ethnic and race-based stress and trauma. London, United Kingdom: Jessica Kingsley

Soojung-Kim Pang, A. (2016). Rest: Why you get more done when you work less. New York, NY: Hachette Book Group.

## Cultural Humility

Cultural Humility: People, Principles and Practices <https://www.youtube.com/watch?v=_Mbu8bvKb_U&list=PL879555ABCCED8B50>

Murray-García, J., & Tervalon, M. (2014). The concept of cultural humility. *Health Affairs (Project Hope)*, *33*(7), 1303.

Murray-García, J., Ngo, V., Marsh, T., Pak, T., Ackerman-Barger, K., & Cavanagh, S. J. (2021). Cultural humility meets antiracism in nurse leader training. *Nurse leader*, *19*(6), 608–615.

Murray-Garcia, J. (2021). A Conversation on Race and Education with Jann Murray-Garcia <https://www.davisvanguard.org/2021/01/a-conversation-on-race-and-education-with-jann-murray-garcia-davis-newest-citizen-of-the-year/>

Tervalon, M., & Murray-García, J. (1998). Cultural humility versus cultural competence: a critical distinction in defining physician training outcomes in multicultural education. *Journal of health care for the poor and underserved*, *9*(2), 117–125.

# Supplement C: Section 4 Accessibility Resources

[12 Tips for Inclusive Teaching](https://mededpublish.org/articles/10-81)

Addy, T.M., Dube, D., Mitchell, K.A., SoRelle, M.E. (2021). What inclusive instructors do: Principles and practices for excellence in college teaching. Sterling, VA: Stylus.

Allarakhia, H. (2022). Strategies for accommodating students with disabilities in higher education. Retrieved from <https://www.facultyfocus.com/articles/equality-inclusion-and-diversity/strategies-for-accommodating-students-with-disabilities-in-higher-education/>

Banks, J. A. (2016). *Cultural diversity and education: Foundations, curriculum, and teaching*.

(6^th^ ed). New York, NY: Routledge.

Gay, G. (2010). *Culturally responsive teaching: Theory, research, and practice* (2nd ed.). New York, NY: Teachers College.

hooks, b. (1994). Teaching to Transgress. New York, NY: Routledge Press.

Howard, T. C. (2010). *Why race and culture matter in schools: Closing the achievement gap in America’s classrooms*. New York, NY: Teachers College.

Ladson-Billings, G. (2009). Just what is critical race theory and what is it doing in a nice field like education? In E. Taylor, D. Gillborn, G. Ladson-Billings, *Critical race theory in education. (pp. 17-36). New York, NY: Routledge.*

Marks, B & Ailey, S. A. (2014) White Paper on Inclusion of Students with Disabilities in Nursing Educational Programs. Sacramento, CA: California Committee on Employment of People with Disabilities (CCEPD). Retrieved from <https://www.aacnnursing.org/Portals/42/AcademicNursing/Tool%20Kits/Student-Disabilities-White-Paper.pdf>

Meeks, L.M. & Jain, N.R. (2018). Accessibility, inclusion and action in medical education: Lived experiences of learners and physicians with disabilities. Retrieved from <https://store.aamc.org/downloadable/download/sample/sample_id/249/>

Meeks, L.M., Jain, N.R., Laird, E.P. (2020). Equal Access for Students with Disabilities: The Guide for Health Science and Professional Education. Springer Publishing Company.

Meeks, L.M. & Neal-Boylan, L. (2020). Disability as Diversity. Springer International Publishing.

National Association of Nurses with Disabilities. <https://nond.org/>

Neal-Boylan, L., Miller, M., & Bell, J. (2018). Building Academic Communities to Support Nursing Students with Disabilities: An Integrative Review. *Building Healthy Academic Communities*, 2(1), DOI: <http://dx.doi.org/10.18061/bhac.v2i1.6342>.

Neal-Boylan, L. & Miller, M. (2017). Treat me like everyone else: The experience of nurses who had disabilities while in school. *Nurse Educator*. DOI: 10.1097/NNE.0000000000000348

Palmer, P.J. (1998). *The courage to teach.* San Francisco, CA: Jossey-Bass.

Singh, S., & Meeks, L. M. (2023). Disability inclusion in medical education: Towards a quality improvement approach. *Medical education*, *57*(1), 102–107. <https://doi.org/10.1111/medu.14878>

Society of Physicians with Disabilities <https://www.physicianswithdisabilities.org/>

University of California Davis (n.d.) Accessibility guide for teaching and presenting. *The Wheel: The Instructional Technology Blog of UC Davis*. <https://wheel.ucdavis.edu/accessibility-guide-teaching-and-presenting>

1. Oxford Languages languages.oup/google-dictionary-en/ [↑](#footnote-ref-1)
