# Supplementary material for: Integrative Curriculum Assessment for Inclusion, Representation, and Equity (I-CAIRE)
Source: MedEdPORTAL. 2025 Feb 28;21:11501. doi: 10.15766/mep_2374-8265.11501 (PMC11868286; doi:10.15766/mep_2374-8265.11501)
Supplement: Supplementary file 1 — I-CAIRE Tool.docxScoring Rubric.docx [file mep_2374-8265.11501-s001.zip › _Educational Summary Report_11501.pdf]

MedEdPORTAL® | The AAMC Journal of Teaching and Learning Resources

Original Publication Open Access

Integrative Curriculum Assessment for Inclusion, Representation, and Equity (I-CAIRE)

Kupiri Ackerman, PhD, RN, CNE*, Elenitsa Sebat, MS, PA-C, Jessica E. Draughon Moret, PhD, RN

*Corresponding author: packerman@ucdavis.edu

Abstract

Introduction: Despite data documenting consistent and ongoing health disparities, health care professionals have yet to make significant change. Health care education that thoughtfully prepares health care providers to address the social determinants of health and health inequities is a crucial step in improving health disparities. The development of an evidence-based tool by which to measure the integration of these concepts within health care education is imperative for achieving this goal. **Methods:** We developed the Integrative Curriculum Assessment for Inclusion, Representation, and Equity (I-CAIRE) tool that assists in evaluating and then integrating health equity concepts into new or existing curricula. The four domains of I-CAIRE include health equity; curriculum content; diversity, inclusion, and representation; and accessibility. A group of eleven experts systematically analyzed the tool, providing feedback to ensure rigor regarding clarity, comprehensiveness, relevancy, validity, and useability. **Results:** The overall mean score across the primary principles and sections was high. After feedback from expert evaluators and with some revisions, all eleven evaluators stated that they were *likely* or *very likely* to recommend the tool. **Discussion:** By conducting a self-assessment of the integration of health equity and social determinants of health content, health care professional schools can identify strengths and areas for improvement. Further, the I-CAIRE tool offers resources that can direct schools to resources for bridging gaps identified in the assessment. I-CAIRE is intended to be a starting point for institutional reflection and discussions that lead to strategic plans to implement these concepts throughout the curricula.

Keywords
Health Equity, Social Justice, Diversity, Equity, Inclusion, Representation, Accessibility, Curriculum Assessment, Cultural Competence, Curriculum Development, Self-Assessment, Editor's Choice

Educational Objectives

By using the I-CAIRE tool, facilitators will be able to:

1. Review curricular standards for health equity within health professions education.
2. Conduct an in-depth evaluation of health professions courses and/or curricula related to health equity, diversity, inclusion, representation, and accessibility.
3. Develop or revise curricula to integrate health equity concepts into health professions programming.

Citation:
Ackerman K, Sebat E, Draughon Moret JE. Integrative Curriculum Assessment for Inclusion, Representation, and Equity (I-CAIRE). *MedEdPORTAL*. 2025;21:11501.
https://doi.org/10.15766/mep_2374-8265.11501

Introduction

The Need for Integrated Assessments of Health Equity
Unsurprisingly, health disparities continue to impact health outcomes reported in the United States. Health disparities are defined as a type of health difference that is closely linked with social, economic, and/or environmental disadvantage that adversely affect groups of people who have systematically experienced greater obstacles to health based on characteristics historically linked to discrimination or exclusion.[1] Health disparity statistics are a crucial first step in addressing the problem; however, simply gathering and reporting data does not improve health outcomes. We must be active in pursuing health equity. Health equity represents an aspirational health care outcome that occurs with the elimination of health inequities and health disparities.[2] It calls for health care professionals to be equity-minded, active, and unrelenting in the pursuit of quality care for all.[3]

Copyright © 2025 Ackerman et al. This is an open-access publication distributed under the terms of the Creative Commons Attribution license.

Integrative Curriculum Assessment for Inclusion, Representation, and Equity (I-CAIRE)

The scholarship related to strategies to mitigate health disparities has evolved. The term cultural congruence was coined by a nursing theorist in the 1960s to describe patient-provider interactions that considered the importance of understanding patients' cultural beliefs related to health care.[4] In the 1980s, that term was expanded upon with the concept of cultural competency, meaning that health care providers should have the knowledge and skill to care for individuals across cultural groups.[5,6] There have been tools developed to assess cultural competency, including the Tool for Assessing Cultural Competence Training[7,8] and the Cultural Competence Model.[5]

In a critique of the limitations of the cultural competency concept, two physicians offered a model of cultural humility that acknowledged the need for skilled health care professionals who could provide care across cultural and identity groups but suggested that lifelong learning and humility were a better approach than seeking competency.[9]

In the last decades, health care scholars have understood that to achieve health equity, providers need to not only have knowledge and skills (competence) and practice with humility, they also need to understand the social determinants of health, be anti-racist, trauma-informed, culturally humble, and see themselves as vital change agents in health care.[3,10-17] The term equity-minded describes the attributes of health care providers who can and will advance health equity.[3]

Given the array of skills that health care providers need to advance health equity, a tool is needed to evaluate and guide health professions schools to graduate equity-minded health care professionals. Because of a gap in the literature related to the scope of these skill sets, we convened an interprofessional group of experts and developed the I-CAIRE tool.

The overarching construct of I-CAIRE is to ensure that health professions schools educate future health care providers to have the knowledge, skill, and desire to advance health equity, as well as the ability see themselves as central to structural changes within the health care system.[3] Health equity has been identified as a primary health care priority, which calls upon schools of health to reimagine curricula in an anti-racist, politically active manner, that addresses health inequities and advances health equity.[17-22] Curriculum evaluation and revision need to be conducted to ensure that our health care education prepares health care providers to conduct patient assessments and to make clinical decisions that include data about social determinants of health and take into consideration historical, structural, and contextual information about patients, families, and communities.[18]

When health profession schools seek to include health equity concepts into their existing curricula, they often attempt to weave them into already established classes. Although weaving may make theoretical sense, operationally, concepts can be added reactively[23] or such that they are only superficially covered and/or quietly drift out of the curriculum. Another strategy is to develop stand-alone health equity or social determinants of health courses. Stand-alone courses can be a great way to build foundational knowledge. However, if these topics are taught alone without being reinforced in other courses, students may perceive, either consciously or not, that their school or program does not truly value health equity.

A hybrid of these two approaches would include a course that focuses on the fundamentals of social determinants of health, health inequities, and health equity with concepts, with these concepts then integrated at critical touch points throughout the remainder of the curriculum. Landry suggested that, during curriculum development or revision, educators can:

> Integrate health equity content longitudinally and in relation to other topics. For example, consider maternal mortality... Lack of content integration further distances clinicians from underlying social contexts that affect patients' health status. An overarching goal should be to eliminate views of health equity and medicine as separate.[10]

The I-CAIRE tool highlights course/curricular core content and a quality improvement process that faculty can include to graduate equity-minded care professionals ready to promote health equity. In total, we included 13 content areas recommended by the National Academies of Sciences, Engineering, and Medicine as essential educational content in health professions to meet the challenge of addressing the social determinants of health, advancing health equity, and improving population health.[17] However, this should not be considered an exhaustive list of topics. Indeed, depending on specialty area, clinical environments, and patient populations served, additional topics may be necessary.

How material is taught is as important as what is taught. We recommend periodic review of learning materials to ensure they reflect diversity, are inclusive, and portray positive and accurate representations of communities of people. In addition,

Copyright © 2025 Ackerman et al. This is an open-access publication distributed under the terms of the Creative Commons Attribution license.

it is important to ensure that teaching and course materials (PowerPoints, cases, textbooks, exams, learning management systems, etc.) resonate with a broad range of learners.[24]

In the accessibility section of the I-CAIRE tool, we focus on both equity pedagogy and students with diversABILITIES. Equity pedagogy refers to teaching in such a way that maximizes opportunities for all learners, no matter background or way of processing information, to engage with the material and be successful in their academic pursuits.[24] There are many differing abilities that have often been called disabilities. The term diversABILITIES reframes how we think about abilities and values the skills and perspectives that people with diverse abilities bring to the learning environment and workplace. Our learning environments should default to optimizing access to learning for all our students.

In sum, the purpose of the I-CAIRE tool is to provide an evidence-based mechanism to conduct an in-depth evaluation of health professions courses and/or curricula focused on health equity, diversity, inclusion, representation, and accessibility. However, simply evaluating curricula is not enough. Faculty and schools must be willing to revise curricula to ensure that it integrates concepts that will help graduate equity-minded health care professionals.

Methods

Structure of I-CAIRE

We developed I-CAIRE (Appendix A) to help educators integrate health equity concepts into curricula rather than fit or add health equity concepts into it. Within the I-CAIRE tool, we developed the following domains: (a) health equity; (b) curriculum content; (c) diversity, representation, inclusion; and (d) accessibility. We prioritized health equity because addressing health disparities and promoting health equity is a health care priority. Specific curricula were included based upon recommendations from the National Academies of Sciences, Engineering, and Medicine and to provide concrete examples of some of the subjects that are integral for promoting health equity. Diversity is a driver of academic and institutional excellence. Diverse health professions teams may be better equipped to solve complex health problems and address health disparities because they tend to be interested in solving health inequities and bring a wealth of knowledge and perspectives to the problem-solving process.[25] However, to leverage the power of diversity, we must also be inclusive and representative. Therefore, we included a section that addresses approaches to diversity, inclusion, and representation. Finally, we included the accessibility section to ensure that teaching and course materials are accessible to a broad range of learners.

Analysis of I-CAIRE Tool

The University of California, Davis, Institutional Review Board determined that this project did not constitute human research. Expert evaluators were provided with a 75-dollar gift certificate to thank them for their time and effort.

We used the expert heuristic evaluation approach as a research design. Heuristic evaluation is one of the most used methods for analyzing usability. This method consists of a group of experts who systematically analyze a system (in this case an evaluation tool), considering a set of explicitly stated design principles (heuristics).[26] The design principles for this study included clarity, comprehensiveness, relevancy, validity, and usability.

The first iteration of the tool was evaluated by three educational experts within UC Davis Health for initial reaction to relevancy and usability. We made a round of revisions based on their feedback. We then had two doctoral students and an assistant professor beta test the I-CAIRE tool on five separate courses that included theory and clinical courses. From the beta testing we learned that the length of time to complete the assessment varied from 1.5-6 hours. We knew that if it took 6 hours to evaluate a course this would be prohibitive, and we therefore revised recommendations about how to use the tool, which were reflected in the second iteration.

We then recruited 11 expert evaluators via email using convenience and snowball sampling. All evaluators were health care faculty who either held a leadership position in or had made scholarly contributions to diversity, equity, and inclusion (to evaluate comprehensiveness and validity), were program leaders with expertise in curriculum design (to evaluate clarity and relevancy), or were faculty actively engaged in curriculum or program review (to evaluate usability). In addition to recruiting based on expertise, our goal was to represent different racial ethnic backgrounds, geographic locations, and health professions within the expert panel. Our recruitment resulted in eight women and three men. Four were Black, one was Arab-American, one was White, two were White-Hispanic, one was White/Indigenous/Mexican-American, one was mixed-race Native American and White, and one was Vietnamese. The following states were represented along with Washington, DC: California, Nevada, Oregon, Connecticut, Georgia, Maryland, and Minnesota. There were four registered nurses, three medical doctors, one psychologist, two physician associates, and a pharmacist.

We asked expert evaluators to analyze the four sections of the I-CAIRE tool using a 5-point Likert scale ranging from *very high level* to *very low level* to assess the four design principles.

Copyright © 2025 Ackerman et al. This is an open-access publication distributed under the terms of the Creative Commons Attribution license.

Operational definitions of each design principle were provided for evaluators (Appendix B). For each of these areas, we provided space for free-text comments about strengths and weaknesses. Finally, we asked for open-ended comments about I-CAIRE (Appendix B). The steps of analysis for the I-CAIRE tool are shown in the Figure.

Results

The overall mean score across the four design principles was 4.2 on a 5-point Likert scale (1 = *very low level*, 5 = *very high level*). We looked at disaggregated scores across the four design principles and I-CAIRE sections evaluating both mean scores, and we also looked at any score by an expert that rated an area below a *high level* (4.0).

Clarity was as defined as the degree to which the section was clearly defined and understandable. The overall mean score for clarity across sections was 4.2. Evaluators provided feedback on terminology that was not clear to them. For each of these, we included additional words and/or concepts in the glossary.

Comprehensiveness was defined as the degree to which the section included the primary concepts related to it. The mean score for comprehensiveness across the four sections of the tool was 4.1. There were some sections where evaluators suggested that we include additional material. In response to evaluator's suggestions, we added material to each of the sections and to the glossary.

Relevancy was the degree to which the survey items in that section were appropriate and connected to the concepts. Regarding relevancy, all respondents rated the four sections of the I-CAIRE tool as *highly relevant* or *very highly relevant*, with a mean score of 4.4. Therefore, this area was not revised or changed.

Validity was defined as the degree to which the section captured or measured the concepts related to it, as defined at the beginning of the section. For the areas of validity there was a mean score of 4.1. In this area we redesigned our rating categories and clarified the terms integrative, strong, and minimal as an evaluative measurement.

We also asked expert evaluators to address usability and whether they would recommend the tool. The usability score was 4.0. We made major revisions to the format of I-CAIRE tool to improve usability. Because we received feedback specifically related to our initial scoring system, we removed this feature and replaced it with rating categories. In addition, expert evaluators recommended we include guidance related to actions that would assist I-CAIRE users in improving their course or curriculum. Therefore, we added extensive resources for them in the supplement section of the I-CAIRE tool.

Finally, we asked expert evaluators if they would recommend the I-CAIRE tool. Initially 10 of 11 stated that they were *likely* or *very likely* to recommend the tool. For the evaluator who stated *maybe*, we asked them to review the tool after the final revisions, and they approved the changes and affirmed that they would now recommend the tool. Expert evaluator scores are shown in the Table.

Discussion

Health disparities are documented across all medical specialties in the United States, making the implementation of change crucial to achieving health equity. The integration of the social determinants of health and health equity into existing curricula is paramount to health professions learners' ability to recognize disparities and implement changes in the health care setting.[10] As health professions schools seek to uphold their mission to mitigate health disparities and to promote health equity, there is a need for tools and assessments that provide a starting place and a standard by which to gauge current and future efforts.

We developed the I-CAIRE tool so health care educational institutions could identify opportunities for growth and recognize achievements in four identified domains: (1) health equity; (2) curriculum content; (3) diversity, inclusion, and representation; and (4) accessibility. The expert evaluation of the I-CAIRE tool indicates that I-CAIRE is sound in terms of clarity, comprehensiveness, relevancy, validity, and usability. Further, there is evidence that the I-CAIRE tool has the potential to improve educational outcomes in schools of health. This tool should be implemented when developing new or

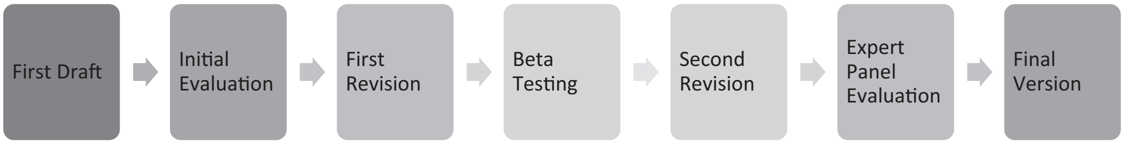

Figure. Steps of analysis of the I-CAIRE tool.

Copyright © 2025 Ackerman et al. This is an open-access publication distributed under the terms of the Creative Commons Attribution license.

Table. Expert Evaluator Scores

Category[a]	Score
Mean score	4.2
Clarity	4.2
Comprehensiveness	4.1
Relevancy	4.4
Validity	4.1
Usability	4.0

[a]Rated based on a 5-point Likert scale (1 = *very low level*, 2 = *low level*, 3 = *fair level*, 4 = *high level*, 5 = *very high level*).

revising existing courses or curricula, or simply to review recommendations for curricular standards related to health equity.

Support for the I-CAIRE at the institutional level will create the most fertile environment for thoughtful, proactive implementation integrated across processes as opposed to simply adding another to-do to faculty and educational designers' respective punch lists.[27-29] This support can be embedded into the overall mission and vision of the institution, whether health equity is mentioned specifically or whether it is implied through a mission and vision that emphasizes patient and community health outcomes.[30,31] Institutional support for implementing I-CAIRE can also take the form of centralized coordination for curriculum assessment and revision.[31,32] When incorporated as a reaction to an event or solely for accreditation, efforts to evaluate and revise courses to address I-CAIRE domains are less likely to be sustainable. In terms of generating buy-in from the intended organization, we recommend focusing on the strengths of the organization and opportunities to provide the best possible care to those the organization serves. The I-CAIRE tool includes recommendations about how to get started.

In terms of broad curriculum revision across health professions, it is our hope that this tool will inspire organizations to get started and then share what constituted challenges and what some potential best practices might be. Whenever possible, integrating adoption and deployment of I-CAIRE into existing processes or re-envisioning and reforming processes to integrate I-CAIRE will assist institutional leaders and educators in streamlining revisions and new assessments.[30,31,33] We envision a few key pathways for implementation of I-CAIRE into schools of health. First, when developing new courses and/or educational programs, including or addressing the elements outlined in the four domains of the I-CAIRE will facilitate a broad representation of diverse perspectives within the new courses. Second, when revising a curriculum—already a large undertaking—carefully and thoughtfully integrating I-CAIRE domains within the revision process allows time to bring faculty, staff, and students together rather than create division. Similarly, if other curriculum assessments are indicated or desired, we recommend incorporating I-CAIRE to benefit from the institutional energy and human capital already being expended in service to the existing assessment. Finally, in terms of continuous quality improvement, we recommend developing an agreed upon cadence for course assessment. Others have recommended a full course review every 2-3 years,[32] whereas waiting for accreditation (5-10 years) could exacerbate an already stressful time. An example of continuous quality improvement would be a self-assessment by the course faculty on an annual or semiannual basis (similar to content expert reviews of specialty area health professions content for remaining current in clinical contexts).[29] As another example, if a course is underperforming in any metric, the need for assessment and revision could present an opportunity to incorporate I-CAIRE elements from the four domains. Even incremental growth in facilitating diverse perspectives within a course can improve student retention as an intermediate outcome, and the terminal outcome of the care alumni provide in their future careers.[17,18,21,34]

It is unlikely that any one tool can serve as a comprehensive assessment or that an assessment tool can undo the inequities or inequalities that exist within an organization or school. However, self-assessment tools can catalyze institutional self-reflection and discussions that lead to both strategic and action plans that focus on advancing health equity.

Although the I-CAIRE tool was evaluated by a panel of experts, we need longitudinal data from schools who avail themselves of the I-CAIRE tool to understand the extent to which the tool offers insight and guidance for curriculum evaluation. A next step is to disseminate the I-CAIRE broadly. Our goal is to encourage implementation of I-CAIRE in diverse health professions education settings so that we may collectively provide further validation for the tool (establish that it is measuring what we think it is measuring) and establish reliability (ensure that the tool produces similar results) across partner universities, health professions, and geographic locations. We anticipate reaching out to and partnering with health professions schools across the country to determine the most effective ways to implement I-CAIRE, develop strategies to catalyze productive conversations across institutional levels and target end users, and make collective progress in meaningful curriculum change related to health equity.

Based on our results, I-CAIRE is a sound tool for self-assessment of various health professional programs. Performing a curricular assessment using the I-CAIRE can help spark dialogue within the

Copyright © 2025 Ackerman et al. This is an open-access publication distributed under the terms of the Creative Commons Attribution license.

institution and encourage real-time solutions that will serve as a nidus of change for our future clinicians and eventually to our patients.

Appendices

A. I-CAIRE Tool.docx

B. Scoring Rubric.docx

All appendices are peer reviewed as integral parts of the Original Publication.

Kupiri Ackerman, PhD, RN, CNE: Associate Dean for Student Affairs and Health Equity, Diversity, and Inclusion, and Professor, Betty Irene Moore School of Nursing, University of California, Davis; ORCID: https://orcid.org/0000-0002-1247-1796

Elenitsa Sebat, MS, PA-C: Assistant Professor and Doctoral Candidate, Betty Irene Moore School of Nursing, University of California, Davis

Jessica E. Draughon Moret, PhD, RN: Associate Professor, Betty Irene Moore School of Nursing, University of California, Davis

Acknowledgments
We would like to acknowledge and thank Amelia Lawless and Marla Shauer for beta testing I-CAIRE. We would also like to acknowledge and thank our expert evaluators for the time and attention they provided to ensure this tool is a meaningful assessment tool for examining and improving health equity content in health professions courses and curricula.

Disclosures
None to report.

Funding/Support
Research reported in this publication was supported by the Gordon and Betty Moore Foundation (Grant Agreement #4294).

Ethical Approval
The University of California, Davis, Institutional Review Board reviewed this project.

References

1. Health equity in Healthy People 2030. US Department of Health and Human Services Office of Disease Prevention and Health Promotion. Accessed April 5, 2022. https://health.gov/healthypeople/priority-areas/health-equity-healthy-people-2030
2. Jones CP. Systems of power, axes of inequity: parallels, intersections, braiding the strands. *Med Care*. 2014;52(10)(suppl 3):S71-S75. https://doi.org/10.1097/MLR.0000000000000216
3. Ackerman-Barger K. Advancing health equity: the rise of equity-minded nurses. Campaign for Action. September 26, 2022. Accessed February 11, 2025. https://campaignforaction.org/the-rise-of-equity-minded-nurses/
4. Leininger MM, ed. *Culture Care Diversity and Universality: A Theory of Nursing.* National League for Nursing Press; 1991.
5. Campinha-Bacote J. A model and instrument for addressing cultural competence in health care. *J Nurs Educ*. 1999;38(5): 203-207. https://doi.org/10.3928/0148-4834-19990501-06
6. Cross TL, Bazron BJ, Dennis KW, Isaacs MR. *Towards a Culturally Competent System of Care: A Monograph on Effective Services for Minority Children Who Are Severely Emotionally Disturbed.* Georgetown University Child Development Center; 1989.
7. Lie D, Boker J, Crandall S, et al. A revised curriculum tool for assessing cultural competency training (TACCT) in health professions education. *MedEdPORTAL*. 2009;5:3185. https://doi.org/10.15766/mep_2374-8265.3185
8. Tool for Assessing Cultural Competence Training (TACCT). Association of American Medical Colleges. Accessed February 11, 2025. https://www.aamc.org/what-we-do/equity-diversity-inclusion/tool-for-assessing-cultural-competence-training
9. Tervalon M, Murray-García J. Cultural humility versus cultural competence: a critical distinction in defining physician training outcomes in multicultural education. *J Health Care Poor Underserved*. 1998;9(2):117-125. https://doi.org/10.1353/hpu.2010.0233
10. Landry AM. Integrating health equity content into health professions education. *AMA J Ethics*. 2021;23(3):E229-234. https://doi.org/10.1001/amajethics.2021.229
11. Ackerman-Barger K, Barksdale D, Draughon Moret JE. Call to action: academic nursing's role in redesigning health care to reduce the human and financial cost of health inequities. *Creat Nurs*. 2023;29(4):360-366. https://doi.org/10.1177/10784535231216466
12. McNair TB, Bensimon EM, Malcom-Piqueux L. *From Equity Talk to Equity Walk: Expanding Practitioner Knowledge for Racial Justice in Higher Education*. Wiley; 2020.
13. Braveman P, Arkin E, Orleans T, Proctor D, Acker J, Plough A. What is health equity? *Behav Sci Policy*. 2018;4(1):1-14. https://doi.org/10.1353/bsp.2018.0000
14. Castrucci BC, Auerbach J. Meeting individual social needs falls short of addressing social determinants of health. *Health Affairs* blog. January 16, 2019. Accessed February 11, 2025. https://www.healthaffairs.org/do/10.1377/hblog20190115.234942/full/
15. Balch B. Curing health care of racism: Nikole Hannah-Jones and Ibram X. Kendi, PhD, call on institutions to foster change. Association of American Medical Colleges. November 17, 2020. Accessed February 11, 2025. https://www.aamc.org/news-insights/curing-health-care-racism-nikole-hannah-jones-and-ibram-x-kendi-phd-call-institutions-foster-change

Copyright © 2025 Ackerman et al. This is an open-access publication distributed under the terms of the Creative Commons Attribution license.

16. Tarleton C, Tong W, McNeill E, Owda A, Barron B, Cunningham H. Preparing medical students for anti-racism at the bedside: teaching skills to mitigate racism and bias in clinical encounters. *MedEdPORTAL*. 2023;19:11333. https://doi.org/10.15766/mep_2374-8265.11333

17. National Academies of Science, Engineering, and Medicine. *The Future of Nursing 2020-2030: Charting a Path to Achieve Health Equity*. National Academies Press; 2021. Accessed February 11, 2025. https://doi.org/10.17226/25982

18. Ackerman-Barger K, London M, White D. When an omitted curriculum becomes a hidden curriculum: let's teach to promote health equity. *J Health Care Poor Underserved*. 2020; 31(4)(suppl):182-192. https://doi.org/10.1353/hpu.2020.0149

19. Bell B. White dominance in nursing education: a target for anti-racist efforts. *Nurs Inq*. 2021;28(1):e12379. https://doi.org/10.1111/nin.12379

20. Crear-Perry J, Maybank A, Keeys M, Mitchell N, Godbolt D. Moving towards anti-racist praxis in medicine. *Lancet*. 2020;396(10249):451-453. https://doi.org/10.1016/S0140-6736(20)31543-9

21. Denizard-Thompson N, Palakshappa D, Vallevand A, et al. Association of a health equity curriculum with medical students' knowledge of social determinants of health and confidence in working with underserved populations. *JAMA Netw Open*. 2021;4(3):e210297. https://doi.org/10.1001/jamanetworkopen.2021.0297

22. Nickitas DM, Emmons KR, Ackerman-Barger K. A policy pathway: nursing's role in advancing diversity and health equity. *Nurs Outlook*. 2022;70(6)(suppl 1):S38-S47. https://doi.org/10.1016/j.outlook.2022.03.013

23. Paul MJ. Double-loop diversity: applying adult learning theory to the cultivation of diverse educational climates in higher education. *Innov High Educ*. 2003;28(1):35-47. https://doi.org/10.1023/A:1025463501820

24. Banks JA. *Cultural Diversity and Education: Foundations, Curriculum, and Teaching*. 6th ed. Routledge; 2016.

25. Swartz TH, Palermo AGS, Masur SK, Aberg JA. The science and value of diversity: closing the gaps in our understanding of inclusion and diversity. *J Infect Dis*. 2019;220(suppl 2):S33-S41. https://doi.org/10.1093/infdis/jiz174

26. Nielsen J. *How to Conduct a Heuristic Evaluation*. Publisher unknown; date unknown. Accessed February 11, 2025. https://www.ingenieriasimple.com/usabilidad/HeuristicEvaluation.pdf

27. Thibault GE. The future of health professions education: emerging trends in the United States. *FASEB Bioadv*. 2020;2(12):685-694. https://doi.org/10.1096/fba.2020-00061

28. Doubeni CA, Fancher TL, Juarez P, et al. A framework for transforming primary care health care professions education and training to promote health equity. *J Health Care Poor Underserved*. 2020;31(4)(suppl):193-207. https://doi.org/10.1353/hpu.2020.0150

29. Charania NAMA, Patel R. Diversity, equity, and inclusion in nursing education: strategies and processes to support inclusive teaching. *J Prof Nurs*. 2022;42:67-72. https://doi.org/10.1016/j.profnurs.2022.05.013

30. Blanchet Garneau A, Browne AJ, Varcoe C. Drawing on antiracist approaches toward a critical antidiscriminatory pedagogy for nursing. *Nurs Inq*. 2018;25(1):e12211. https://doi.org/10.1111/nin.12211

31. Browne AJ, Varcoe C, Lavoie J, et al. Enhancing health care equity with Indigenous populations: evidence-based strategies from an ethnographic study. *BMC Health Serv Res*. 2016;16:544. https://doi.org/10.1186/s12913-016-1707-9

32. Goldman EF, Swayze SS, Swinehart SE, Schroth WS. Effecting curricular change through comprehensive course assessment: using structure and process to change outcomes. *Acad Med*. 2012;87(3):300-307. https://doi.org/10.1097/ACM.0b013e318244739c

33. McLemore MR. Using retrofit, reform, and reimagine to advance toward health equity. *J Perinat Neonatal Nurs*. 2022;36(2): 99-102. https://doi.org/10.1097/JPN.0000000000000639

34. Beard KV. Examining the impact of critical multicultural education training on the multicultural attitudes, awareness, and practices of nurse educators. *J Prof Nurs*. 2016;32(6):439-448. https://doi.org/10.1016/j.profnurs.2016.05.007

Received: June 4, 2024
Accepted: December 23, 2024
Published: February 28, 2025

Copyright © 2025 Ackerman et al. This is an open-access publication distributed under the terms of the Creative Commons Attribution license.
